# Unveiling the dynamics of circulating tumor cells in colorectal cancer: from biology to clinical applications

**DOI:** 10.3389/fcell.2024.1498032

**Published:** 2024-10-30

**Authors:** Claudia Dompé, Aleksandra Chojnowska, Rodryg Ramlau, Michal Nowicki, Catherine Alix-Panabières, Joanna Budna-Tukan

**Affiliations:** ^1^ Department of Immunology, Poznan University of Medical Sciences, Poznan, Poland; ^2^ Doctoral School, Poznan University of Medical Sciences, Poznan, Poland; ^3^ Department of Oncology, Poznan University of Medical Sciences, Poznan, Poland; ^4^ Department of Histology and Embryology, Poznan University of Medical Sciences, Poznan, Poland; ^5^ Laboratory of Rare Human Circulating Cells and Liquid Biopsy (LCCRH), University Medical Centre of Montpellier, Montpellier, France; ^6^ Centre de Recherche en Ecologie et Evolution du Cancer, Maladies Infectieuses et Vecteurs: Ecologie, Génétique, Evolution et Contrôle, University of Montpellier, Centre National de la Recherche Scientifique, Institut de Recherche Pour le Dévelopement, Montpellier, France; ^7^ European Liquid Biopsy Society (ELBS), Hamburg, Germany; ^8^ Department of Anatomy and Histology, Collegium Medicum, University of Zielona Gora, Zielona Gora, Poland

**Keywords:** circulating tumor cells, colorectal cancer, metastasis, liquid biopsy, cancer cell biology

## Abstract

This review delves into the pivotal role of circulating tumor cells (CTCs) in colorectal cancer (CRC) metastasis, focusing on their biological properties, interactions with the immune system, advanced detection techniques, and clinical implications. We explored how metastasis-competent CTCs evade immune surveillance and proliferate, utilizing cutting-edge detection and isolation technologies, such as microfluidic devices and immunological assays, to enhance sensitivity and specificity. The review highlights the significant impact of CTC interactions with immune cells on tumor progression and patient outcomes. It discusses the application of these findings in clinical settings, including non-invasive liquid biopsies for early diagnosis, prognosis, and treatment monitoring. Despite advancements, challenges remain, such as the need for standardized methods to consistently capture and analyze CTCs. Addressing these challenges through further molecular and cellular research on CTCs could lead to improved interventions and outcomes for CRC patients, underscoring the importance of unraveling the complex dynamics of CTCs in cancer progression.

## 1 Introduction

Colorectal cancer (CRC) ranks among the most diagnosed malignancies worldwide, with a substantial impact on morbidity and mortality (Siegel et al., 2023). Despite advances in treatment modalities, metastatic spread remains a significant challenge, contributing to disease progression and therapeutic resistance. In recent years, the study of circulating tumor cells (CTCs) has emerged as a promising avenue for unraveling the intricacies of metastatic dissemination and refining clinical management strategies (Alix-Panabières and Pantel, 2016).

Metastasis, a complex process driven by cellular and molecular interactions, hinges on the dissemination of cancer cells, with CTCs marking a critical stage in systemic spread (Castro-Giner and Aceto, 2020). Investigating the biology of metastasis-competent CTCs sheds light on the mechanisms driving tumor dissemination. Furthermore, the interplay between CTCs and the immune system unveils a dynamic battlefield within the host microenvironment, wherein immune surveillance and evasion mechanisms dictate the fate of disseminated tumor cells. Navigating this intricate interplay between CTCs and immune cells holds immense significance in deciphering the determinants of immune evasion, tumor immune escape, and potential immunotherapeutic interventions in CRC (Leone et al., 2018).

Technological advancements have facilitated the precise enrichment, identification, and characterization of CTCs, offering insights into their molecular signatures. This translation of CTC research has revolutionized CRC management, with CTCs serving as prognostic biomarkers and guiding treatment decisions in real-time, ushering in a new era of personalized medicine. However, challenges persist, including technical limitations and the need for standardization in CTC analysis.

In this paper, we comprehensively explore the dynamics of CTCs in CRC, spanning their biology, immune interactions, technological advancements, clinical applications, and existing limitations. We provide a holistic understanding of CTC research through an integrative approach to inform future advancements in CRC diagnosis, prognosis, and treatment.

## 2 Biology of metastasis-competent CTCs

CTCs constitute a heterogeneous population of cancer cells that have detached from a primary tumor and contribute to the spread to the metastasis sites. CTCs have a median half-life of 1–2.5 h and are present in the circulation as individual cells or clusters, which is believed to establish an increased metastatic ability (Meng et al., 2004). Their phenotypic aspect varies between cancer type and stage of disease; these changes are usually associated with different prognoses (Parkinson et al., 2012). Their presence is associated with shorter progression-free survival (PFS) and overall survival (OS) in CRC (Cohen et al., 2009). Notably, CTCs are more prevalent in aggressive diseases, which makes them instrumental in monitoring cancer evolution and assessing response to therapy (Barbazan et al., 2014).

**FIGURE 1:**
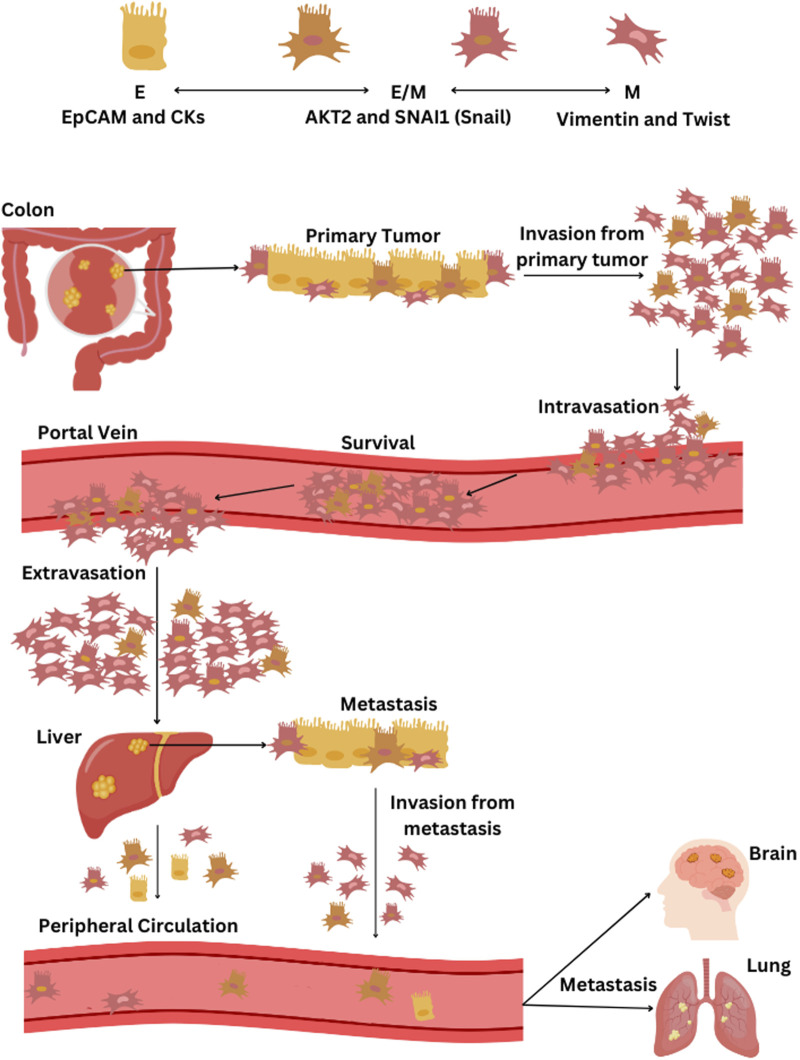
The process of EMT and the dissemination of CTCs from CRC through the bloodstream. CTC phenotypes are characterized by specific markers: epithelial (E) phenotype by EpCAM and cytokeratins (CKs), epithelial-mesenchymal hybrid (E/M) phenotype by AKT2 and SNAI1 (Snail), and the mesenchymal (M) phenotype by vimentin and twist. The progression of CRC and the spread of CTCs via the bloodstream begins with the primary tumor in the colon, primarily composed of epithelial cells, followed by tumor cell invasion into surrounding tissues and intravasation into blood vessels. CTCs that detach from the tumor, predominantly of the M- and some E/M phenotypes, then circulate in the portal vein. Most CTCs that survive in the portal vein are of the M-phenotype and eventually extravasate in the liver, leading to potential liver metastasis. Some CTCs enter the peripheral circulation; they predominantly present weaker E/M and E-phenotypes and may initiate a further metastatic cascade that can potentially spread to other organs, including the lungs and brain. If liver metastasis occurs, cells may detach from the secondary tumor, undergo once more EMT transition, and contribute to the further metastatic cascade towards other organs.

The CTC blood concentration is extremely low, and metastatic patients could have 1–10 CTCs per mL of blood, making the isolation step complicated (Domínguez-Vigil et al., 2018). While most research has concentrated on detecting and counting CTCs in peripheral blood, fewer studies have explored the possibility that capturing CTCs may be more effective in vessels nearer to the tumor. Tumor-proximal liquid biopsies enhance CTC diagnostic capabilities because drawing blood from a tumor-draining vein significantly boosts the likelihood of capturing CTCs freshly shed by the tumor (Buscail et al., 2019). Clinical studies showed that the liver filters against viable CTCs when cancer cells transit through the portal vein, and only a smaller amount of CTC enters the peripheral circulation (Figure 1) (Denève et al., 2013). Jiao et al. reported that CTC counts before surgery were elevated in the portal circulation and hepatic vein compared to peripheral blood (Jiao et al., 2009). Furthermore, Wind et al. demonstrated that in patients undergoing surgery for primary colon cancer, the count of CTCs notably rose during the operation and was significantly greater in portal blood compared to peripheral blood samples (Wind et al., 2009). CTC isolation is a complex process that allows the study of cellular contents and gene information expression, facilitating functional analysis of CTC cultures, genomic characterization, and protein analysis (Pantel and Speicher, 2016).

CTC isolation involves three different steps: enrichment, detection, and cell characterization. The enrichment step increases CTC concentration based on the identification of biological, like protein markers, or physical properties, such as size and density (Alix-Panabières and Pantel, 2016). The detection step employs immune-cytologic, molecular, or functional assays. The CTC characterization can be performed at the single cell level at different levels: at the genomic, transcriptomic, proteomic, and secretomic levels. High heterogeneity is attributed to their omics characteristics (Micalizzi et al., 2017). The functional characterization of CTC may employ establishing CTC cultures, lines, xenografts, and genotyping (Cayrefourcq et al., 2015; Baccelli et al., 2013). The diversity of CTC markers underscores CTC heterogeneity among different cancer types and even within a patient. Studies highlighted that different organ microenvironments can influence the types of CTCs detected. This heterogeneity results from spatially different blood microenvironments and temporal changes in cancer stage and therapy response (Venesio et al., 2018). For example, *EGFR* (epidermal growth factor receptor) and *KRAS* (Kirsten rat sarcoma virus) mutations are crucial for guiding treatment and diagnosis in CRC patients. However, these are not always reliable. Concordance rates for KRAS mutations between CTCs and primary tumors vary, with differences attributed to CTC selection protocols (Buim et al., 2015). Furthermore, defining the entire CTC population using limited molecular markers is challenging. For example, EpCAM (Epithelial cell adhesion molecule) is an epithelial marker widely used in cancer diagnosis but is limited in EpCAM-negative or low-expression tumors (Pantel et al., 2012). Specifically, CTCs undergoing Epithelial Mesenchymal Transition (EMT) may downregulate epithelial markers, impacting detection rates. Using both epithelial and mesenchymal markers, or marker-independent methods, improves isolation efficiency (Sleijfer et al., 2007). Further details on isolation techniques will be discussed later.

The process of metastasis formation related to CTCs begins when these invade the surrounding tissue through the lymphatic vessels or the bloodstream at any stage of tumorigenesis, survive in the circulation, evade immunosurveillance, and can extravasate into a tissue, and finally colonize new tissue or organ (Steeg, 2006). CTCs’ metastatic capacity is enhanced by their migratory competence; in CRC, CTC-specific transcriptome profiling highlighted 410 CTC-specific genes associated with cell movement, adhesion, as well as regulation of cell death and proliferation (Barbazán et al., 2012). CTCs may employ a mechanism to avoid cell death when tumor cells are not attached but travel in the bloodstream. This mechanism indicates another CTC hallmark called « anoikis resistance,» marked by the expression of TNFRSF1B (tumor necrosis factor receptor superfamily member 1B) and BCL11A (B-cell lymphoma/leukemia 11A) (Zhang et al., 2016).

Furthermore, the EMT process supports their metastatic potential, which enhances their ability to extravasate into the bloodstream (Alix-Panabières et al., 2017). EMT’s dual need to improve the motility and invasiveness of cancer cells within the primary tumor and MET for the ultimate step of metastatic colonization in distant organs indicates the dynamic interplay between EMT and MET. CTCs that underwent partial EMT display a hybrid state that allows adaptation to a more aggressive phenotype (Eslami-S et al., 2023). A study conducted by Soler et al. indicated that colon CTC lines are tumor cells with an intermediate EMT phenotype, they maintained a stable epithelial phenotype, and they expressed markers associated with both epithelial and mesenchymal characteristics (Soler et al., 2018). Another study showed that CTCs derived from a colon cancer patient exhibit an intermediate EMT profile with high plasticity, downregulated EMT, and upregulating MET markers (Balcik-Ercin et al., 2021). Ultimately, these results suggest that CTCs possess mesenchymal features for migration and intravasation but maintain an epithelial state.

Studies underscore the rarity of CRC-derived CTCs in peripheral blood due to this unique metastatic pathway, with a significant portion being filtered or retained by the liver, which is often the site of initial metastases (Figure 1). Mesenteric venous blood compartments of CRC patients harbor more CTCs than peripheral blood (Kaifi et al., 2015). The shift in the EMT status of CTCs from portal to peripheral blood indicates that mesenchymal traits in CTCs could enhance their retention in liver tissue in cases of liver cancer (Sun et al., 2018). M-CTCs are associated with more aggressive forms of tumors (Zhao et al., 2019). A previous study demonstrated that more significant quantities of M-CTCs in portal blood than in peripheral blood correlate with worse outcomes (Dong et al., 2020). Furthermore, colon CTC lines exhibited stem-cell features (Soler et al., 2018; Balcik-Ercin et al., 2021). This combination of EMT phenotype and stem-cell characteristics further enhances their potential for dissemination and metastasis. The exchange of CTC phenotypes persists even after liver metastasis when cells dissociate from the secondary tumor and initiate an additional metastatic cascade that can potentially spread to other organs, including the lungs and brain (Figure 1) (Hanssen et al., 2018).

CTCs’ interactions with blood and tissue components, including clusters with different cell types, are fundamental for cancer survival and the promotion of metastatic disease. For example, bidirectional interactions between tumor cells and platelets are crucial for cancer progression, with cohesive CTC aggregates thought to have enhanced invasive potential (Yu et al., 2021). These interactions involve direct cell-cell contacts, the release of proteins, and the formation of tumor-platelet aggregates. The reciprocal influence between cancer cells and platelets leads to alterations in both cell types. For example, exposure of platelets to CRC cells results in the production of extracellular vesicles that inhibit tumor growth but promote metastasis (Eslami-S et al., 2023). Furthermore, the intricate interplay between tumor-associated macrophages (TAMs) and cancer cells contributes to the EMT program of CTCs, facilitating their intravasation into the bloodstream (Wei et al., 2019). The observed fusion of macrophages and tumor cells underscores a potential mechanism for immune evasion and invasion.

Overall, CTCs are a highly heterogeneous population that employs different molecular pathways to survive in the bloodstream. A better understanding of their biology and functioning may ease us into early cancer detection and assess therapy effectiveness.

## 3 Navigating the interplay between CTCs and the immune system

**FIGURE 2:**
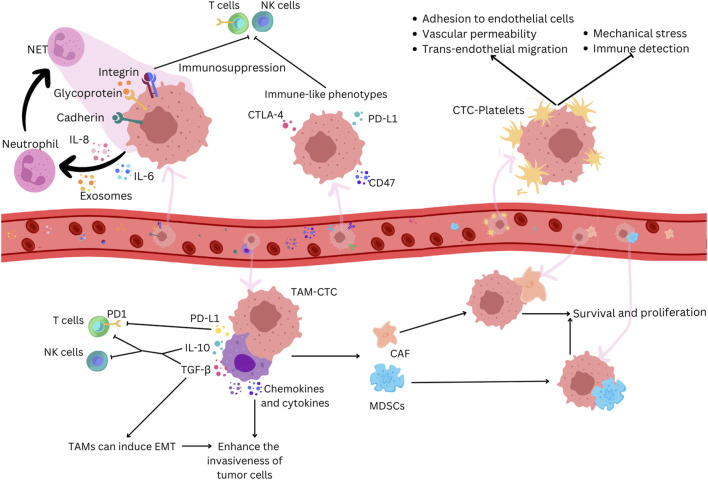
CTCs adopt immune-like phenotypes to evade immune recognition in cancer. Neutrophils can promote tumor growth by suppressing anti-tumor immune responses and facilitating tumor cell proliferation and metastasis. CTCs secrete chemokines that attract neutrophils, which, when activated, promote the formation of NETs which trap CTCs by creating a physical mesh that immobilizes them in the bloodstream and by facilitating adhesion through interactions involving a series of cell adhesion proteins, such as cadherin, integrin, and surface glycoprotein. NETs can suppress immune surveillance by inhibiting the activation of NK cells and effector T cells. CTCs express immune antigens like CTLA-4 and CD47 to evade immune responses. Platelets bind to CTCs, protecting them from mechanical stress and immune surveillance and facilitating their adhesion to endothelial cells, increasing vascular permeability and supporting trans-endothelial migration. TAMs interact with CTCs, secreting factors that help them evade immune detection by T cells and NK cells and induce EMT. TAMs produce chemokines and cytokines that enhance the invasiveness of tumor cells, and their interaction with CTCs attracts MDSCs and CAFs. These cells enhance CTC survival and proliferation through the secretion of chemokines and cytokines that further suppress immune responses.

In the bloodstream, CTCs face various stresses during peripheral circulation, including shear stress, loss of anchorage, and interactions between cytokines and immune cells (Figure 2). To restore cellular homeostasis, CTCs might activate adaptive stress response pathways that increase stress tolerance and significantly contribute to their phenotypic heterogeneity (Mohme et al., 2017). CTCs employ different ways to survive these unfavorable conditions and to avoid immune surveillance. The molecular profiles of tumors and associated cells suggest that cancer cells can develop immune-like phenotypes, expressing immune antigens such as PDL1 (Programmed death-ligand 1), CD47 (cluster of differentiation 47), and CD14 to achieve immune resistance (Raimondi et al., 2020). This strategy involves interactions with pro-tumorigenic cells, evasion from lymphocyte cell-mediated immune responses, and production of immune mediators, creating a tumor-supportive environment (Steinert et al., 2014). The mechanisms CTCs develop to evade antitumor immune responses and to make a compromised microenvironment involve the interaction with various blood components such as neutrophils, macrophages, platelets, myeloid-derived suppressor cells (MDSCs), and cancer-associated fibroblasts (CAFs) to escape the immune system and promote survival.

Neutrophils are associated with cancer progression, and an increase in their amount is linked with poor prognosis (Zhang et al., 2017). CTCs increase the expression of immunosuppressive chemokines like chemokine (C-C motif) ligand 5 (CCL5) and C-X-C motif chemokine 5 (CXCL5). These chemokines recruit regulatory T cells (Tregs) and neutrophils, suppressing the antitumor immune response (Sun et al., 2021). CTCs within CTC-neutrophil clusters express granulocyte colony-stimulating factor (G-CSF) and other cytokines that stimulate neutrophil recruitment (Szczerba et al., 2019). Neutrophils can directly adhere to CTCs through interactions like Mac-1/ICAM-1 (Macrophage-1 antigen/Intercellular Adhesion Molecule 1) or VCAM1 (Vascular cell adhesion protein 1) dependent intercellular junctions and involve cytokine-receptor crosstalk with IL-1β (interleukin-1 beta) and IL-6, promoting extravasation and proliferation (Szczerba et al., 2019; Spicer et al., 2012). Neutrophil extracellular traps (NETs) can capture CTCs in circulation, promoting metastatic dissemination (Cools-Lartigue et al., 2013). NETs, formed by activated neutrophils, trap CTCs by creating a physical mesh that immobilizes them in the bloodstream and by facilitating adhesion through interactions involving a series of cell adhesion proteins, such as cadherin, integrin, and surface glycoprotein (Chen et al., 2018). NETs can suppress immune surveillance by inhibiting the activation of peripheral leukocytes, natural killer (NK) cells, and effector T cells and cooperating with other immune cells (Kumagai et al., 2020).

The interaction between CTCs and tumor-associated macrophages (TAMs) plays a crucial role in various stages of metastasis (Cortese et al., 2019). Studies observe that CTCs can induce the differentiation of monocytes into TAMs, leading to the secretion of mediators that promote leukocyte recruitment, migration, and invasion (Hamilton and Rath, 2017). In colorectal cancer, a feedback loop between TAMs and cancer cells is essential for the EMT program of CTCs and their entry into the bloodstream (Wei et al., 2019). TAMs contribute to CTCs’ mechanical adhesiveness, endurance, and the formation of protective cell clusters, conferring resistance to shear stress (Osmulski et al., 2021). Macrophage-tumor cell hybrids, exhibiting M2-like macrophage phenotypes and epithelial markers, have been identified in the blood of patients with colorectal cancers (Manjunath et al., 2020). When transplanted into mice, these hybrids spread widely and form lesions in distant tissues, correlating with disease stage and overall survival (Manjunath et al., 2020). Understanding the direct interaction and molecular fusion between CTCs and macrophages is crucial for identifying potential therapeutic targets.

The interaction between CTCs and platelets significantly influences cancer metastasis and progression; it involves various receptors and ligands and influences processes such as extravasation and metastasis. Platelets form aggregates with CTCs in the bloodstream, facilitated by prothrombotic microparticles or tissue factor expression by CTCs (Thomas et al., 2015). Platelets protect CTCs from mechanical stress, induce resistance to anoikis, and hinder NK cell attack through various mechanisms (Placke et al., 2009; Egan et al., 2014; Haemmerle et al., 2017). Platelet-released extracellular vesicles (EVs) accelerate the EMT process, promoting invasion and metastasis (Plantureux et al., 2020). Platelets also play a role in adhesion to endothelial cells, supporting firm adherence of CTCs to the endothelial wall (Schumacher et al., 2013). Additionally, platelet-secreted factors contribute to increased vascular permeability, facilitating trans-endothelial migration of tumor cells (Foss et al., 2020). Recent research suggests potential antitumor strategies, including using specific agents to block tumor-specific platelet functions and reverse the immunosuppressive tumor microenvironment (Xu et al., 2020).

MDSCs compose a diverse group of myeloid cells with immunosuppressive properties that contribute to metastatic dissemination in cancer. A study revealed a significant expansion of polymorphonuclear (PMN)-MDSCs across various cancer types compared to infection and inflammation (Cassetta et al., 2020). Clusters formed by CTCs and MDSCs are believed to evade T-cell immune surveillance (Liu et al., 2016). *In vitro* co-culture experiments demonstrated that CTCs from melanoma and breast cancer patients, when exposed to PMN-MDSCs, enhanced the production of reactive oxygen species in CTCs, promoting their proliferation (Sprouse et al., 2019).

CAFs are abundant in the tumor microenvironment and play crucial roles in tumor initiation, angiogenesis, metastasis, and drug resistance (Chen and Song, 2019). Studies revealed that CAFs remodel the extracellular matrix, facilitating tumor invasion and communication with cancer cells through the secretion of growth factors, chemokines, and cytokines (Gaggioli et al., 2007). CAFs can form complexes with CTCs in the bloodstream, where CTCs are either attached to or surrounded by CAFs, promoting dissemination and invasion (Agarwal et al., 2018). The interplay between CAFs and CTCs revealed that CTCs can transport CAFs from the primary tumor to metastatic sites. Depleting CAFs reduces the number of metastases and improves survival in experimental models (Duda et al., 2010). CAFs within the complexes provide a supportive microenvironment for CTCs, promoting their survival and protecting them from immune surveillance and apoptosis. CAFs also offer protection to CTCs against fluid shear forces during dissemination. In a three-dimensional co-culture model, CAFs induced shear resistance in prostate tumor cells through stable intercellular contact and soluble factors associated with cell survival, invasion, and EMT (Ortiz-Otero et al., 2020).

The ongoing dialogue between malignant cells and their microenvironment progresses into a cooperative interaction, helping to evade antitumor immune responses and create a tumor microenvironment permissive for metastasis. Tumors often activate immune checkpoint pathways to resist antitumor immune responses. Immune checkpoint molecules such as PD-L1, CTLA-4, and CD47 were identified as key players in tumor immune evasion, contributing to tumor growth and metastasis (Aktar et al., 2023a). The predictive value of CTCs varies across different tumor stages, suggesting a correlation between the innate immunity and acquired immune evasion mechanisms of CTCs and their metastatic potential. Discrepancies and similarities in PD-L1 expression between tumor tissue and CTCs highlight the importance of assessing peripheral and local immunity for comprehensive insights (Koh et al., 2019). Dysregulated expressions of KRAS, p53, and CTLA-4 (cytotoxic T-lymphocyte-associated protein) contribute to tumor-immune system crosstalk and can influence the expression of immune checkpoint molecules. For example, loss of p53 function enhances PD-L1 surface expression, resulting in T-cell inactivation (Kortlever et al., 2017). Oncogenic events such as KRAS mutations have also been implicated in suppressing or evading anti-tumor immune responses through immune checkpoint molecules (Cohen et al., 2008). A correlation between KRAS and CTLA-4 mRNA expression in CTCs and primary tumor tissues was observed, suggesting a potential role of CTLA-4 in KRAS-mediated CRC progression (Aktar et al., 2023b).

## 4 Cutting-edge technologies for the enrichment, identification, detection, and isolation of CTCs

**FIGURE 3:**
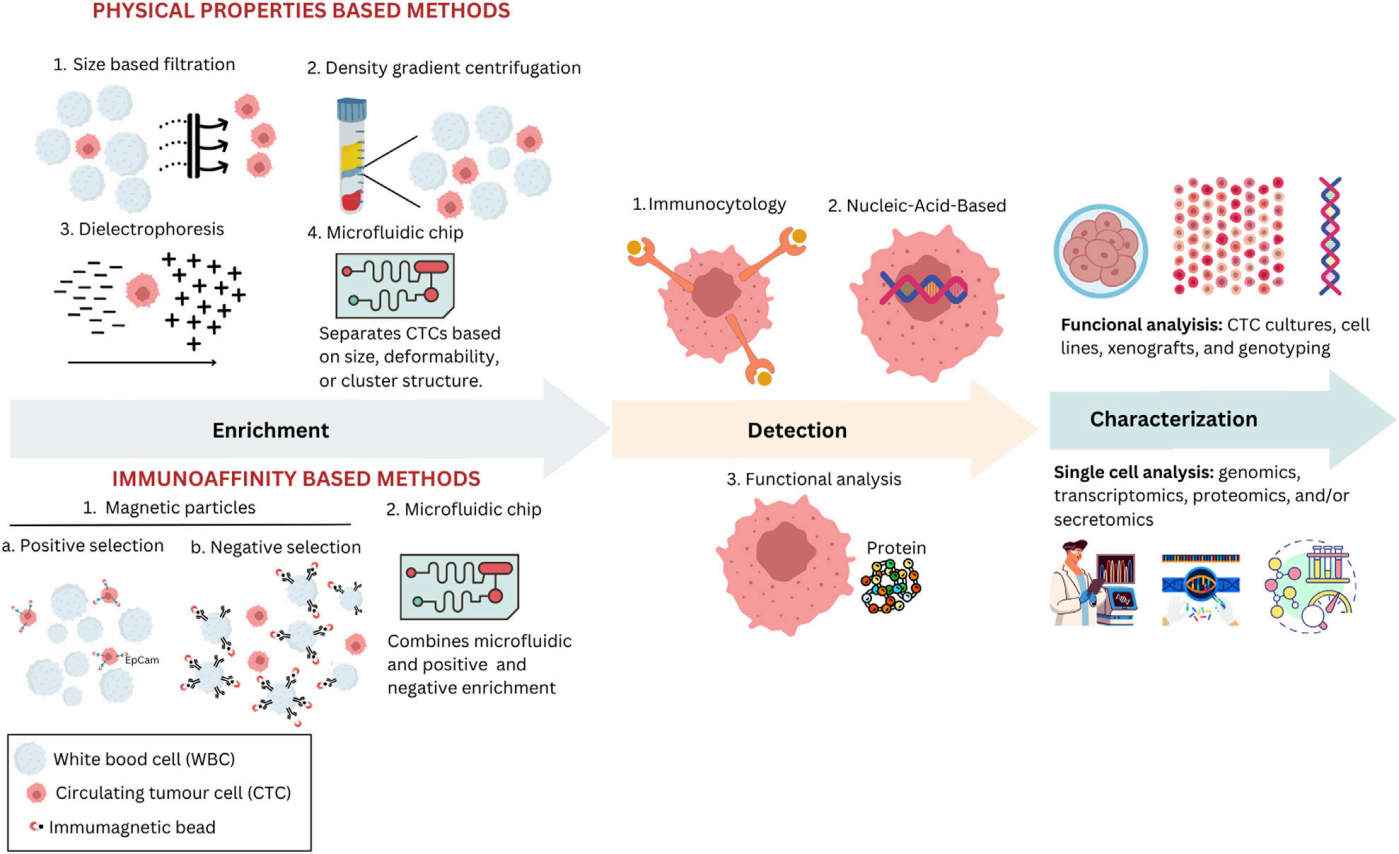
Various methodologies are used to enrich, detect, and characterize CTCs in clinical and research settings. Physical properties-based methods include size-based filtration, density gradient centrifugation, dielectrophoresis, and microfluidic chips, each utilizing unique physical characteristics such as size and electric properties to isolate CTCs. In immunoaffinity-based methods, magnetic particles are employed either for positive selection using specific antibodies or for negative selection to remove unwanted cells, enhancing the purity of CTCs; microfluidic chips can also employ one of these selection strategies. After enrichment, isolated CTCs are identified through various methods, including immunocytological, molecular, or functional assays. Immunocytological methods involve staining CTCs using antibodies targeting specific cellular markers. Molecular methods focus on identifying CTCs through nucleic-acid-based assays. Functional assays allow viable CTCs to be detected based on their biological activities, such as assays that identify specific proteins secreted by CTCs. Characterization techniques, such as functional analysis through CTC cultures and single-cell analysis encompassing genomics and proteomics, are used to assess the biological and functional properties of isolated CTCs.

A feature of liquid biopsy is the possibility of collecting the blood sample required for analysis directly from the patient’s home. This method offers faster results than tissue biopsy analysis and can aid in detecting and genetically profiling hidden malignancies in patients without accessible tissue cancer samples. While tissue biopsy remains the primary cancer diagnosis and biomarker assessment method, liquid biopsy has become widely utilized (Rodríguez et al., 2021). Liquid biopsy offers a non-invasive and repeatable approach for profiling cancer genotypes and monitoring patients over time. Various circulating biomarkers can be identified and characterized in blood samples, whether in plasma or serum, such as tumor-derived EVs or exosomes, proteins, circulating free RNA (cfRNA), circulating tumor DNA (ctDNA), and tumor-educated platelets (TEP) (Alix-Panabières and Pantel, 2021). One can identify single CTCs and CTC clusters, circulating endothelial cells (CEC), and CAFs in the cellular fraction. CTCs are present in the blood in very low concentrations and are often found in just 10%–30% of early-stage cancer patients, which makes their isolation and analysis complicated, expensive, and technically complex (Domínguez-Vigil et al., 2018). Their detection usually lacks consistency, raising concerns regarding the reliability of CTC markers and casting doubt on their specificity (Marquette et al., 2020). This low detection rate is further impaired as tumor cells flowing into the bloodstream are quickly trapped by organs they encounter first. In later stages, CTC counts increase when metastases are present in various organs (Suo et al., 2017). New methods for increasing blood volume for CTC analysis are being explored but could lead to false positives in healthy individuals or those with benign inflammatory diseases (Pantel et al., 2012). Therefore, challenges remain in using CTC assays for early cancer diagnosis. CTC isolation requires steps such as enrichment, detection, and cell characterization (Figure 3).

Researchers employ advanced technologies, various platforms, and assays for CTC analysis through cell size recognition or expression of cell-surface antigens (Alix-Panabières and Pantel, 2016). Enrichment is the first step in CTC assays, which increases the concentration of CTCs, making it easier to detect single tumor cells. This step can be based on biological properties, like protein markers, or physical properties, like size, density, deformability, or electric charge density. Combining these principles optimizes CTC yield. Several methods based on physical properties are employed to isolate CTCs. Density gradient centrifugation involves separating blood components based on migration through a medium of varying density under centrifugal force (Jacob et al., 2007). However, challenges include the loss of large CTCs, cytotoxicity, and low purity. Magnetic-activated cell sorting (MACS) employs magnetic nanoparticles attached to antibodies to capture cells (Königsberg et al., 2010). On the other hand, a negative CTC selection strategy involves erythrocyte lysis and magnetic depletion of leukocytes, enhancing viability independent of CTC phenotype. RosetteSep™ (STEMCELL Tech) uses antibodies to form rosettes by crosslinking red and white blood cells, which are then separated from CTCs through centrifugation (Nomura et al., 2023). Microfiltration methods use microscale constrictions to capture cells by size or deformability, allowing rapid enrichment with drawbacks like heterogeneity, membrane clogging, and recovery issues (Lim et al., 2012). Microfluidics manipulates small fluid volumes, separating CTCs based on size, deformability, or cluster structure (Sarioglu et al., 2015). However, heterogeneity leads to CTC loss and white blood cell contamination. Dielectric properties, exploited in ApoStream™ and DEPArray™, use dielectrophoretic force for isolation, offering single-cell recovery and viability, but face limitations like low sample volumes and changes in cell properties over time (Peeters et al., 2013; Gupta et al., 2012).

After enrichment, an identification step is crucial to detect CTCs among surrounding leukocytes. This is achieved through various immune-cytologic, molecular, or functional assays. Immunoaffinity-based methods use affinity reactions between antibodies and specific target antigens on the cells of interest, achieving high recovery and purity rates (Pamme, 2012). This approach allows for single-step detection and isolation of CTCs, while its efficacy depends on antigens’ expression and specificity, immunomagnetic labeling efficiency, and separation technique (Talasaz et al., 2009). A mix of antibodies targeting multiple antigens often addresses the lack of specificity in tumor markers. Two main approaches include direct (positive selection) and indirect CTC capture (negative selection). Positive CTC selection methods target surface tumor antigens, although their heterogeneity makes a universal marker challenging. The CellSearch^®^ system, the most widely utilized, is the sole FDA-cleared assay for CTC detection, isolation, and enumeration (Hofman et al., 2011). This technology is based on the expression of EpCAM on CTCs. However, challenges arise when EpCAM expression decreases, yielding false positives in benign diseases and false negatives during the EMT process (Pantel et al., 2012). Although all colon CTC lines strongly express EpCAM, indicating that the initiation of EMT occurs while EpCAM is still present in CRC, the clinical relevance of using positive selection methods like the CellSearch^®^ remains valid (Eslami-S et al., 2020). The complex functional properties of CTCs further complicate their detection, emphasizing the need for non-EpCAM-based technologies. The EPithelial ImmunoSPOT (EPISPOT) is a functional assay that identifies CTCs after negative selection by detecting specific epithelial proteins released and secreted by them (Denève et al., 2013). Its more sensitive liquid microdroplet format, EPIDROP, allows the enumeration of CTCs at the single-cell level (Pantel and Alix-Panabières, 2019). The AdnaTest analyses the expression patterns on cancer cells using antibody-coated beads specific to the type of cancer and real-time polymerase chain reaction (Andreopoulou et al., 2012). CTCs can be detected through nucleic acid-based approaches. Identifying CTCs at the DNA or mRNA level involves creating PCR assays designed with primers targeting transcripts specific to tissues, organs, or tumors or that detect unique genetic alterations like mutations, translocations, or methylation patterns specific to the tumor (Mastoraki et al., 2018).

Once CTCs are separated, the cells can undergo subsequent analysis. Molecular characterization provides crucial information regarding their origin, dissemination capacity, drug susceptibility or resistance, and transcriptional plasticity. In 2015, Cayrefourcq et al. successfully established the first stable CTC line from a patient with metastatic colon cancer (Cayrefourcq et al., 2015). Additionally, the researchers derived and characterized eight additional cell lines from the same patient at various stages of clinical management, including during treatment and cancer progression (Cayrefourcq et al., 2021). This unique series of CTC lines shed light on the selection process of treatment-resistant clones with distinct phenotypes driving disease advancement. Recent advances in single-cell sequencing technologies, particularly RNA sequencing (RNA-seq), have been essential in studying CTC biology. Transcriptome/RNA profiling can be conducted through sequencing or *in situ* hybridization (Wu et al., 2016). RNA-seq of single CTCs has led to identifying prognostic signatures, metastatic drivers, drug targets, and resistance mechanisms in various cancers (Pan and Jia, 2021).

While CTCs have been detected and investigated across various cancer types, their utility as biomarkers for predicting outcomes, identifying minimal residual disease, guiding therapeutic decisions, and monitoring cancer progression is particularly prominent in breast, prostate, colon, and lung cancer patients. The liquid biopsy assay offers a minimally invasive option with improved sample quality and rapid execution time. It can be repeated multiple times throughout a patient’s oncological journey, capturing the cancer’s spatial and temporal heterogeneity and clonal evolution. Moreover, it demonstrates a high concordance rate with tissue next-generation sequencing (NGS) (Vitiello et al., 2019). In the future, integrated platforms will combine CTC enrichment, detection, and characterization.

## 5 Clinical applications of CTC research in colorectal cancer

Tumor heterogeneity possesses significant challenges in the clinical application of molecular prognostic markers; it contributes to drug resistance, limits precision in histological diagnoses, and hampers the efficacy of many oncology therapies. Existing tumor characterization methods provide a global overview, but metastatic cells in different microenvironments may express different biomarkers. CTCs offer an alternative to cancer diagnostics, allowing the monitoring of dynamic changes in tumor biology more accurately than a single biopsy. Recent advances in CTC research enable reliable quantification and molecular characterization at the single-cell level (Heymann and Téllez-Gabriel, 2018). CTC heterogeneity can reflect selective pressures from therapeutic interventions. Monitoring biomarker changes in CTCs over multiple therapies may reveal insights into tumor evolution and help identify effective drugs for individual patients, especially those with or developing treatment resistance. In CRC, CTCs have great potential as biomarkers for detecting tumors, predicting outcomes, monitoring therapy, and tailoring treatments (Heidrich et al., 2021). However, the use of CTC analysis in CRC is still limited due to challenges in detection and reproducibility. Studies have shown varying detection rates of CTCs in different stages of CRC, ranging from 9% in nodal-negative CRC to 45% in CRC stages I-IV (Abdalla et al., 2021). Identifying genome instability promoters or suppressors in solid tumors could limit tumor evolutionary processes.

Tumor extent and residual status post-treatment are critical predictors of CRC outcome, and detection of CTCs can aid in identifying potential metastases earlier and selecting chemotherapy-resistant patients who may benefit from alternative therapeutic regimens. CTC counts before and during treatment predict PFS and OS, providing valuable prognostic information beyond imaging modalities. CTCs can assess chemotherapy effectiveness and prognosis in CRC patients. Research indicates that identifying CTCs post-chemotherapy predicts outcomes in stage III colon cancer, highlighting potential resistance to standard treatments (Lu et al., 2013). Studies investigating gene expression in CTCs have revealed significant correlations with clinicopathological features in cancer. In CRC, carcinoembryonic antigen (CEA) levels can predict tumor invasion, occult metastasis risk, and relapse rates (McCall et al., 1994). KRAS gene mutations are associated with increased relapse and death risks in CRC patients and confer resistance to EGFR inhibitors (Amado et al., 2023). CTCs carrying wild-type KRAS show longer PFS and OS in treated patients, highlighting their predictive value in treatment response (Yen et al., 2009).

Transcriptome analysis at the single-cell level further enhances the understanding of intra-tumoral heterogeneity and treatment response prediction. Gene expression profiles of CTCs, such as *LGR5* (Leucine-rich repeat-containing G-protein coupled receptor 5) expression, have been linked to metastasis and poor prognosis in CRC patients (Wang et al., 2018). Additionally, multigene expression panels in CTCs can predict treatment response and identify patients who may benefit from specific therapies, allowing for timely adjustments in treatment plans (Insua et al., 2017). Chen et al. found that the oncogene *ECT2* (epithelial cell transforming) expressed by CTCs in peripheral blood was more sensitive than serum CEA levels, particularly in advanced-stage CRC patients, and its expression on CTC surfaces was identified as a potential prognostic marker for CRC (Wang et al., 2019). Another study showed elevated expression levels of *MAGEA1-6* (Melanoma-associated antigen 1) or *hTERT* (human Telomerase reverse transcriptase) genes in CTCs of patients with T3 and T4 stages compared to T1 and T2 stages (Kim et al., 2017). Additionally, Welinder et al. demonstrated that the presence of the *Akt-2* gene in CTCs of CRC patients was associated with shorter PFS and OS (Welinder et al., 2015). The expression of different genes is also used to monitor disease progression and predict outcomes in patients with CRC (Cai et al., 2019).

NGS enables high-throughput molecular diagnosis using CTCs, aiding in understanding immune evasion mechanisms and drug resistance and identifying new signaling pathways in cancer. Specifically, in a study, the detection of specific chromosomal variations and expression of markers like *COX-2* (cyclooxygenase-2) in various phenotypes of CTCs revealed a close association between *COX-2* expression in mesenchymal CTCs and metastasis (Cai et al., 2019). Functional CTC analysis using cell lines and xenograft models has identified potential therapeutic targets, such as the PI3K/AKT/mTOR (phosphoinositide 3-kinases/protein kinase B/mammalian target of rapamycin) signaling pathway (Smit et al., 2020). VEGF (vascular endothelial growth factor) overexpression predicts relapse, and EGFR expression influences treatment response (Tsai et al., 2013). Genetic polymorphisms and microsatellite instability offer treatment response and prognosis insights (Smith et al., 2002).

CTCs are crucial in CRC management, providing valuable insights into disease progression and treatment response. CTC analysis allows for real-time monitoring of treatment efficacy and detection of metastatic relapse and disease progression, complementing traditional approaches focused on primary tumor analysis. Immunocytochemistry, immunohistochemistry, and RT-PCR are commonly used techniques for CTC detection, although standardized technology and conflicting results still need to be improved for routine clinical implementation. Advanced detection methods, such as colorimetric membrane arrays and weighted enzymatic chip arrays (WEnCA), promise to enhance sensitivity and accuracy for early diagnosis and postoperative surveillance in CRC patients (Huang et al., 2016). Integrating CTC analysis into clinical practice can improve personalized treatment strategies and patient outcomes. However, challenges in widespread adoption, including cost considerations and technical expertise, persist. Standardization of blood collection and handling protocols are essential for implementing single-cell analysis, which has shown high clinical and scientific impact in understanding CTC heterogeneity across different cancer types. Despite these challenges, ongoing development and refinement of various techniques hold promise for advancing CRC diagnosis and monitoring, ultimately leading to more effective disease management.

## 6 The Unknown explored: Investigating colorectal cancer through CTCs

Despite advancements in diagnostic and therapeutic methods, challenges persist in detecting and managing CRC, particularly in the early stages. CTCs have garnered attention as potential biomarkers in oncological precision medicine for CRC detection and monitoring, offering non-invasive and potentially informative insights into disease progression and treatment response (Alix-Panabières and Pantel, 2021). The ability to eliminate CTCs during therapy holds promise as a possible endpoint for clinical studies testing new drugs (Heller et al., 2018). While presenting numerous opportunities for innovation and advancement, CTC’s clinical utility still needs to be improved. Liquid biopsy increases costs when used alongside tissue tests and cannot analyze non-DNA biomarkers or provide histopathological and phenotypical information (Marrugo-Ramírez et al., 2018).

The detection of CTCs in CRC faces sensitivity and specificity challenges, which may be attributed to the rapid trapping of tumor cells by organs like the liver, particularly in the early stages (Buscail et al., 2019). As metastases develop in various organs, CTC counts increase. The low abundance of CTCs in peripheral blood and the presence of non-malignant cells contribute to false-negative results, limiting the reliability of CTC detection as a standalone diagnostic tool (Thorsteinsson et al., 2011). On the other hand, new methods to increase blood volume for CTC analysis are being explored but may yield false positives in healthy individuals or those with benign inflammatory diseases (Pantel et al., 2012). Technical limitations in CTC isolation and characterization methods hinder their widespread clinical application. Ongoing advancements in CTC isolation promise to improve CTC detection in CRC, offering the potential to overcome technical hurdles and enhance the clinical utility of CTCs as biomarkers. Beyond their role as biomarkers, CTCs can be utilized for functional assays, such as *ex vivo* drug sensitivity testing (Pallante et al., 2019). This allows researchers to assess the response of an individual patient’s tumor cells to various therapies, guiding personalized treatment decisions and potentially revealing drug resistance mechanisms. Furthermore, culturing CTCs in three-dimensional (3D) models, like tumorspheres and organoids, provides a more accurate representation of the tumor microenvironment. These 3D models enable the study of complex cell-to-cell interactions, metastatic processes, and clonal evolution, ultimately deepening our understanding of cancer biology and facilitating the development of more effective therapies (Pallante et al., 2019). Integrating multi-omic analyses, such as genomic, transcriptomic, and proteomic profiling, with CTC characterization can provide comprehensive insights into CRC biology and heterogeneity (Micalizzi et al., 2017). Combined multi-omic approaches offer the opportunity to elucidate molecular mechanisms driving CRC progression, metastasis, and therapeutic resistance, facilitating personalized treatment strategies.

Efforts to localize primary or micrometastatic tumor lesions using liquid biopsies face challenges due to overlapping marker expressions and changes in transcriptional profiles (Mohamed Suhaimi et al., 2015). CRC is characterized by intratumoral and intertumoral heterogeneity, reflected in the diverse phenotypic and genotypic profiles of CTCs (Jones et al., 2017). The heterogeneity of CTCs poses challenges in accurately capturing the full spectrum of disease biology and may limit their utility as prognostic and predictive biomarkers. Understanding the biological processes governing the release of CTCs from tumors is crucial. Liquid biopsy, encompassing CTCs, ctDNA, and other biomarkers, holds promise for early detection and screening of CRC. Integrating CTC analysis with other liquid biopsy components may enhance the sensitivity and specificity of early CRC detection, enabling timely intervention and improved patient outcomes (Alix-Panabières and Pantel, 2021). Translation of CTC-based assays from research settings to clinical practice requires rigorous validation in large-scale prospective clinical trials. Collaborative efforts involving multidisciplinary teams, industry partners, and regulatory agencies are essential for validating CTC-based assays’ clinical utility, safety, and cost-effectiveness in CRC management. Initiatives like the European Liquid Biopsy Society (ELBS, www.elbs.eu) promote better collaboration and integration among fundamental, translational, and clinical research fields. These collaborations are crucial for the widespread application of CTCs in the clinic.

## 7 Conclusion

In conclusion, studying CTCs in CRC offers valuable insights into metastasis, immune interactions, and treatment efficacy. Integrating technological advancements with our understanding of CTC biology, we foresee a promising future for personalized medicine in CRC. However, challenges such as technical limitations and heterogeneity of CTC populations must be addressed to fully realize the potential of CTCs in clinical practice. By overcoming these obstacles through continued research and innovation, we can enhance patient care and outcomes in CRC.
